# Multi-Sensor Fusion of Infrared and Electro-Optic Signals for High Resolution Night Images

**DOI:** 10.3390/s120810326

**Published:** 2012-07-30

**Authors:** Xiaopeng Huang, Ravi Netravali, Hong Man, Victor Lawrence

**Affiliations:** 1 Department of Electrical and Computer Engineering, Stevens Institute of Technology, Hoboken, NJ 07030, USA; E-Mails: hong.man@stevens.edu (H.M.); vlawrenc@stevens.edu (V.L.); 2 Department of Electrical Engineering, Columbia University, New York, NY 10027, USA; E-Mail: ran2290@gmail.com

**Keywords:** theoretical PSF, inverse filter design, IR image transformation, EO image edge detection, image pair blending/superimposing

## Abstract

Electro-optic (EO) image sensors exhibit the properties of high resolution and low noise level at daytime, but they do not work in dark environments. Infrared (IR) image sensors exhibit poor resolution and cannot separate objects with similar temperature. Therefore, we propose a novel framework of IR image enhancement based on the information (e.g., edge) from EO images, which improves the resolution of IR images and helps us distinguish objects at night. Our framework superimposing/blending the edges of the EO image onto the corresponding transformed IR image improves their resolution. In this framework, we adopt the theoretical point spread function (PSF) proposed by Hardie *et al.* for the IR image, which has the modulation transfer function (MTF) of a uniform detector array and the incoherent optical transfer function (OTF) of diffraction-limited optics. In addition, we design an inverse filter for the proposed PSF and use it for the IR image transformation. The framework requires four main steps: (1) inverse filter-based IR image transformation; (2) EO image edge detection; (3) registration; and (4) blending/superimposing of the obtained image pair. Simulation results show both blended and superimposed IR images, and demonstrate that blended IR images have better quality over the superimposed images. Additionally, based on the same steps, simulation result shows a blended IR image of better quality when only the original IR image is available.

## 1. Introduction

Infrared (IR) imaging systems depend on the thermal contrast between objects in the camera view in order to distinguish them. These systems create images by utilizing the infrared energy emitted by the objects as a result of their temperature difference with the background and emissivity. Unfortunately, such IR image sensors have low resolution and high noise levels. Therefore, accurately distinguishing objects at night is a challenging topic that has perplexed scientists and engineers for a long time. Multi-sensor fusion of IR and electro-optic (EO) images is an effective way to solve this challenge. It combines images from the two sources to obtain a single composite image with improved resolution, low noise, and ability to see clearly at night. Up to now, scientists have developed many efficient image fusion algorithms, such as the expectation maximization (EM) fusing algorithm [1], the discrete wavelet transform (DWT) fusing algorithm [2], and the Laplacian pyramid fusing algorithm [3,4] presents an overview of the recent progress and the current state-of-the-art techniques of color image fusion for night vision applications. Additionally, much literature focuses on presenting techniques for estimating a high-resolution IR image via the optimized IR image system [5–7], e.g., with reduced aliasing. However, based on the existing approaches, it is still impossible to accurately distinguish the edges of different objects at night when they have a similar temperature and background.

In this paper, we propose a novel framework to solve this problem. We use edges of EO images previously recorded during daytime to improve real-time IR imagery at night. Assuming we have a pair of EO and IR images, then four steps are required for this framework: (1) transform the original IR image into a temperature map via the point spread function (PSF) and design an inverse filter from the literature; (2) detect an edge map of the high resolution EO image; (3) register the transformed IR image and the detected edge map; and (4) blend/superimpose the detected edge map of the EO image with the transformed IR image. In this framework, on one hand, we adopt a theoretical PSF for the IR image system, which comprises the modulation transfer function (MTF) of a uniform detector array and the incoherent optical transfer function (OTF) of diffraction-limited optics; on the other hand, we employ indexing superimposing method and linear blending method to complete Step (4), respectively. In addition, we assume both IR and EO sensors capture the same objects. We will show both blended and superimposed IR images, and demonstrate that blended IR images have better quality over the superimposed images. Final simulation results will reveal that with the help of the blended/superimposed edge map, we can distinguish between objects in the transformed IR image and even small parts of a single object. Above all, the performance of this proposed framework is independent of objects' temperature. That is to say, if objects have similar temperature, it's very difficult for us to distinguish edges of objects in IR images based on traditional image enhancement methods. However, in our proposed framework, the edges are detected from EO images, so we could distinguish objects independent of their temperature. In summary, the proposed framework can provide important aspects of vision at night.

In particular, we compare the results generated by the use of EO and IR images to those generated by the use of only an IR image. The following questions will be addressed in this paper: (1) Are there some things we can see now that we could not see before by utilizing traditional image fusion methods? (2) Do blended IR images have better quality over the superimposed images? Or *vice versa*? (3) How do the results look if we use only IR images for processing? Can we derive a similar image with only IR images? (4) Are there things we can only see partially with only IR images? (5) Are there “false alarms”, e.g., things that should not be seen that are now seen falsely? Could this include some extraneous patterns? (6) In summary, what are the improvements in picture quality using our framework?

The remainder of this paper is organized as follows: Section 2 introduces the IR image transformation process in terms of the proposed theoretical PSF and designed inverse filter. Section 3 presents the edge detection of EO images and the related image registration process. Simulation results are provided in Section 4. Finally, some conclusions and future work are addressed in Section 5.

## 2. IR Image Transformation

### 2.1. Theoretical PSF

We adopt the theoretical PSF of Hardie *et al.* [8] for IR image systems, which is based on the MTF of a uniform detector array and the incoherent OTF of diffraction-limited optics. The adopted PSF in this paper is only an example, any other infrared sensors could be adopted to conduct the same test and simulation, which may have better performance. Even we could do research on optimization of parameter settings of PSF in terms of different applications/requirements.

The primary contributor is the finite detector size, and this effect is spatially invariant for a uniform detector array. We begin by considering an infrared system with this uniform detector array. We can model the effect of the integration of light intensity over the span of the detectors as a linear convolution operation with a PSF determined by the geometry of a single detector. We assume an isoplanatic model for optics.

Let d(x, y) denote the PSF. Applying the Fourier transform to d(x, y) yields the effective continuous frequency response resulting from the detectors [8]:

(1) D(u,v)=FT{d(x,y)}

where FT{·} stands for the continuous Fourier transform. In addition, define the incoherent OTF of the optics to be H_0_ (u, v), where u and v are the horizontal and vertical frequencies measured in cycles/mm. The overall system OTF is given by the product of these, yielding [8]:

(2) $$\text{H}(\text{u},\text{v})=\text{D}(\text{u},\text{v}){\text{H}}_{0}(\text{u},\text{v})$$

Then, the overall continuous system PSF is given by [8]:

(3) $${\text{h}}_{\text{c}}(\text{x},\text{y})={\text{FT}}^{-1}\{\text{H}(\text{u},\text{v})\}$$

where FT^−1^ represents the inverse Fourier transform.

**Figure 1.**
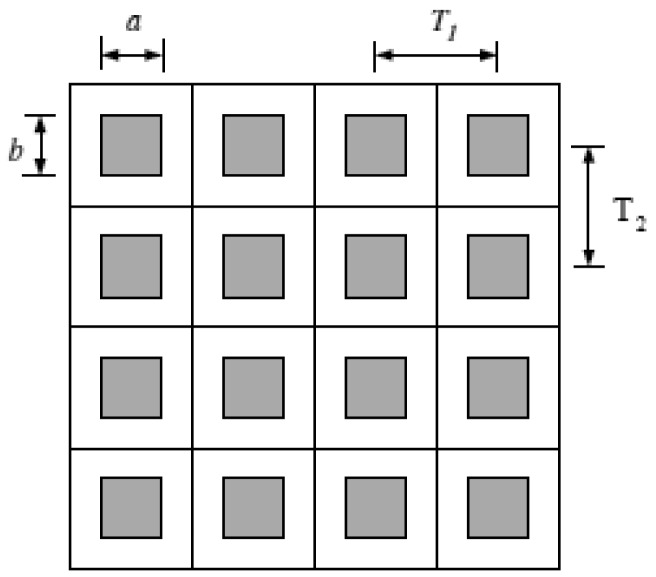
Critical dimensions of the uniform detector array [8].

In this paper, we consider an IR system with a uniform rectangular detector array, which is illustrated in Figure 1, where a and b are the active region dimensions measured in millimeters (mm) and T1 and T2 are the horizontal and vertical sample spacings. The shaded areas represent the active region of each detector. In this case, the detector model PSF is given by [9]:

(4) $$\text{D}(\text{x},\text{y})=\frac{1}{\text{ab}}\text{rect}\left(\frac{\text{x}}{\text{a}},\frac{\text{y}}{\text{b}}\right)=\{\begin{array}{c} 1,\;\text{for}\;\left|\frac{\text{x}}{\text{a}}\right|<\frac{1}{2}\;\text{and}\;\left|\frac{\text{x}}{\text{b}}\right|<\frac{1}{2}\\ 0,\;\text{otherwise} \end{array}$$

The corresponding effective continuous frequency response resulting from the detector is given by [8]:

(5) $$\text{D}(\text{u},\text{v})=\sin\text{c}(\text{au},\text{bv})=\frac{\sin(\pi \text{au})\sin(\pi \text{bv})}{\pi^{2}\text{aubv}}$$

The incoherent OTF of diffraction-limited optics with a circular exit pupil can be found as [8]:

(6) $${\text{H}}_{0}(\text{u},\text{v})=\{\begin{array}{c} \frac{2}{\pi }[\cos^{-1}\left(\frac{\rho }{\rho_{\text{c}}}\right)-\frac{\rho }{\rho_{\text{c}}}\sqrt{1-{\left(\frac{\rho }{\rho_{\text{c}}}\right)}^{2}}\;\text{for}\;\rho <\rho_{\text{c}}\\ 0\;\text{otherwise} \end{array}$$

where $\rho =\sqrt{{\text{u}}^{2}+{\text{v}}^{2}}$. The radial system cutoff frequency ρ_c_ is given by [8]:

(7) $$\rho_{\text{c}}=\frac{1}{\lambda \text{f}/\#}$$

where f/# is the f-number of the optics and λ is the wavelength of light considered. Since the cutoff frequency of the optics H_0_ (u, v) is ρ_c_, so is also the cutoff frequency of the overall IR system's OTF.

**Figure 2.**
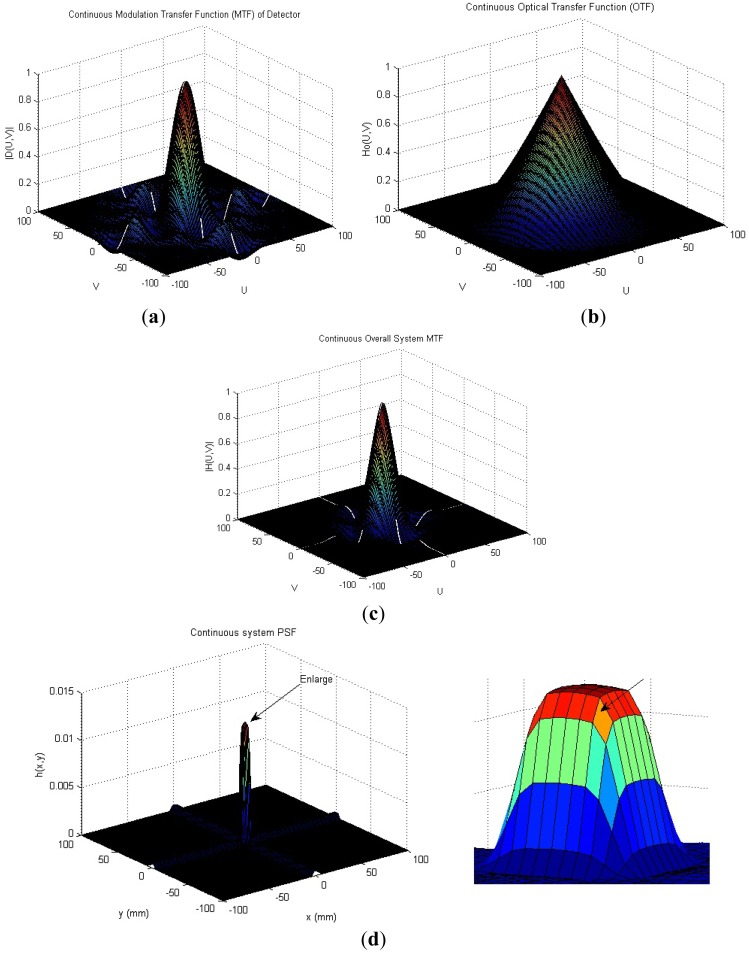
FLIR imaging system. (**a**) Effective MTF of the detectors, |D (u, v)|; (**b**) Diffraction-limited OTF for the optics, H (u, v); (**c**) Overall system MTF; (**d**) Continuous system PSF (enlarged PSF on right side).

We consider a particular IR imaging system as an example; the typical system considered is the forward-looking infrared (FLIR) imager. In order to make our adopted PSF realistic, parameter settings of this PSF are the same as in [8]. This system has square detectors of size a = b = 0.040 mm, the imager is equipped with 100 mm f/3 optics, the center wavelength = 0.004 mm and the cutoff frequency 83.3 cycles/mm is used for the OTF calculation. In Figure 2, Figure 2(a) shows the effective MTF of the detectors, |D (u, v)|, and Figure 2(b) shows the diffraction-limited OTF for the optics, H (u, v). The overall system MTF is shown in Figure 2(c), and the continuous system PSF is shown in Figure 2(d).

### 2.2. Inverse Filtering and IR Image Transformation

Usually direct inverse filtering is the simplest approach we can take to restoring a degraded image, which ignores the noise term in the model and forms an estimator in the form of [10]:

(8) $$\hat{\text{F}}(\text{u},\text{v})=\frac{\text{G}\;(\text{u},\text{v})}{\text{H}\;(\text{u},\text{v})}$$

where G (u, v) is the degraded image and H (u, v) is the system PSF. Then, we obtain the corresponding estimate of the image by taking the inverse Fourier transform of F̂ (u, v).

**Figure 3.**
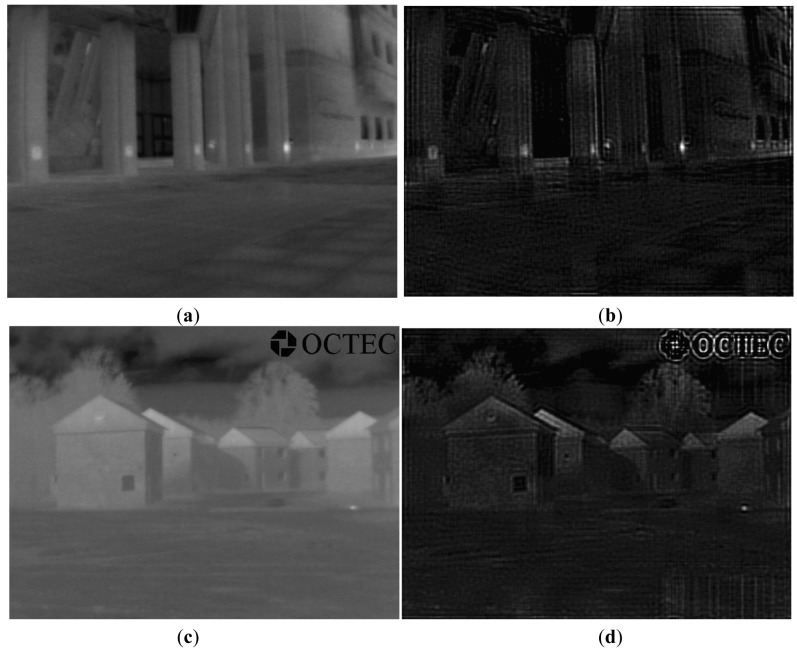
Examples of IRE image transformation using the designed inverse filter. (**a**) Original IR Image 1; (**b**) Transformed IR Image 1 via the inverse filter; (**c**) Original IR Image 2; (**d**) Transformed IR Image 2 via the inverse filter.

In this paper, we assume the proposed theoretical PSF to be H (u, v) in the designed inverse filter. If we let the original IR image pass through the designed inverse filter, then we can obtain a transformed IR image with temperature information of the objects. Figure 3 shows two IR image transformation examples using the designed inverse filter.

## 3. Image Edge Detection and Registration

### 3.1. Image Edge Detection

Image edge detection refers to the process of identifying and locating sharp two-dimensional discontinuities in an image. The discontinuities are abrupt changes in pixel intensity that characterize boundaries of objects in a scene. By far, edge detection is the most common approach for detecting meaningful discontinuities in intensity values. There are many edge operators to perform edge detection, e.g., Sobel, Prewitt, Roberts, Laplacian of a Gaussian (LoG), Zero crossings and Canny. So far the Canny edge detection algorithm [11] has been known as the optimal edge detector. The detailed description of the Canny edge detector can be found in [11], and the syntax for the Canny edge detector in Matlab is:

(9) [g,t]=edge(f,‘canny’,T,sigma)

where f is the original EO image, T is a vector, T = [T1, T2], containing the two thresholds of the preceding procedure, and sigma is the standard deviation of the smoothing filter. We can change these parameters so as to produce clean edge maps. Detected edges have different performance (e.g., cleanness) with different scales. Our selection criteria of clean edges are: (1) detected edges should cover at most of objects; (2) detected edges should be continuous curves; (3) detected edges should not contain any unnecessary information (e.g., not main edges of objects).

**Figure 4.**
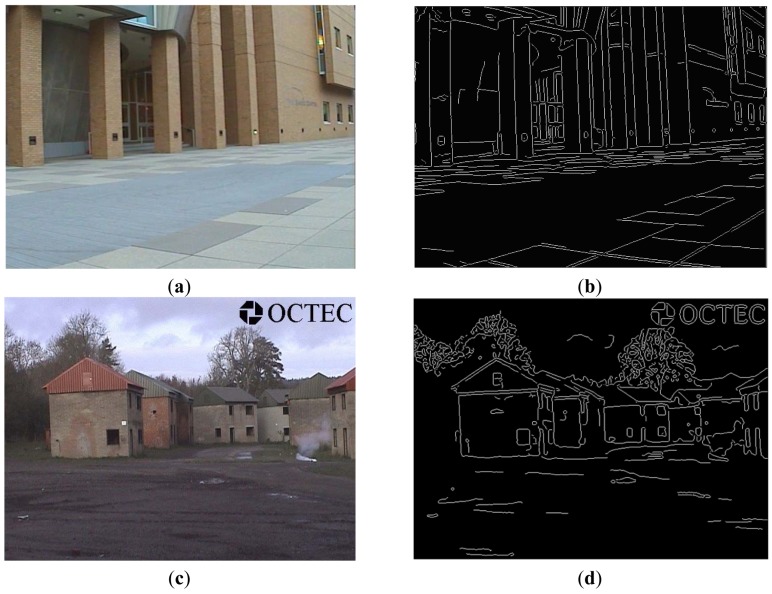

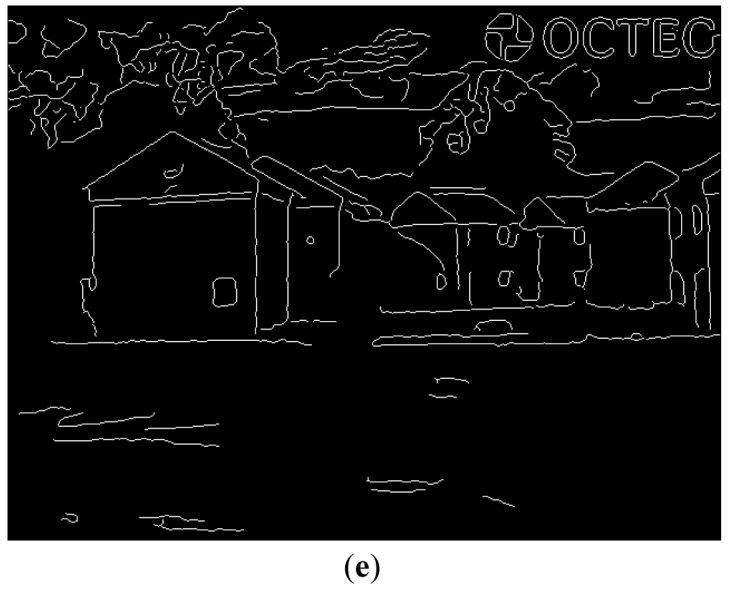
Two edge detection results of EO images via the Canny edge operator. (**a**) Original EO Image 1; (**b**) Detected edge of EO Image 1; (**c**) Original EO Image; (**d**) Detected edge of EO Image 2; (**e**) Detected edge of IR Image 2.

Figure 4 shows two edge detection results of EO images via the Canny edge operator, where sigma = 1, T1 = 0.04 and T2 = 0.09. Observed from the obtained results, we can find out that the edge-detected images are clean, and results cover all main edges of objects in original EO images.

### 3.2. Image Pair Registration

Image registration methods, which seek to align two or more images of the same scene, generally consist of the following basic steps: (1) detect features; (2) match corresponding features; (3) infer geometric transformation; and (4) use the geometric transformation to align one image with the other. In this paper, we adopt the cp2tform function from the Matlab image processing toolbox to do the manual image pair registration. The more complicated image registration processes (e.g., image registration involves shift, rotation and transformation), are beyond the scope of our paper. In some particular cases, we could complete a partial image registration rather than the whole image registration. We could even adopt other methods to help obtain the exactly aligned images (e.g., image warping).

## 4. Blended/Superimposed Image Results

For the last step, the contour map of the EO image is mapped onto the corresponding IR image. We show results of both directly superimposed and seamlessly blended image pairs, and compare the performance difference between these two approaches.

### 4.1. Superimposed Image Results

One approach of the last step of our proposed framework is to superimpose the detected edge of the EO image onto the transformed IR image [12]. Typically, there are two basic ways to superimpose images. One involves using transparency to overlay images, and the other involves indexing the image data to replace pixels. Here, we adopt the indexing approach to achieve our purpose.

**Figure 5.**
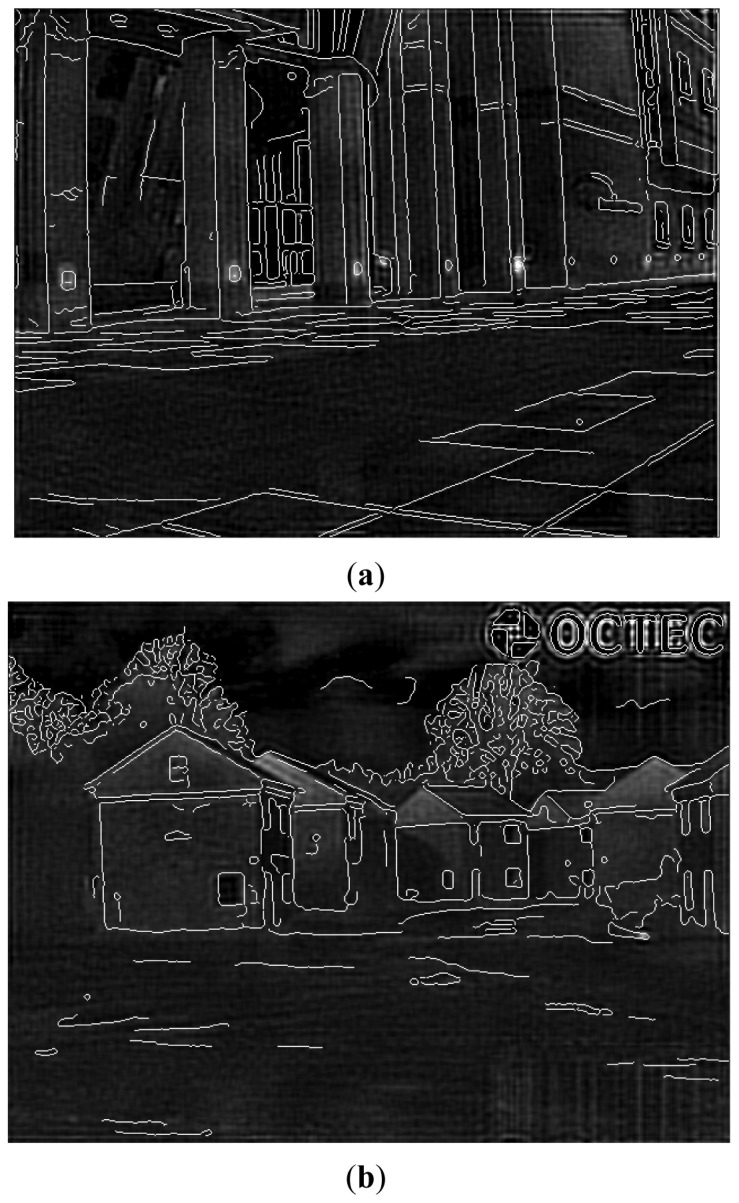
Examples of superimposed images. (**a**) Indexing superimposed result of the transformed IR Image 1; (**b**) Indexing superimposed result of the transformed IR Image 2.

Figure 5 shows two superimposed examples. Generally speaking, based on our results, we can distinguish objects accurately at night via the clean edge of the EO image superimposed on the corresponding transformed IR image.

### 4.2. Blended Image Results

The last step of our framework is to blend the edge-detected EO image with the transformed IR image and the original IR image [13]. On the one hand, in order to make the blended edge seamlessly match with its corresponding object, we let the edge-detected image pass through a small size low pass filter (2 by 2 mean filter). On the other hand, in order to make sure the blended edge is not prominent in the blended image, we set the pixel value of the detected edge to be 0.4 rather than its default value 1. We only want to observe edges of objects rather than change original structure of IR images, so the weight of blended edges is small.

In this paper, we adopt the alpha blending process [14] to achieve the fourth step. Two images before blending are read in the variable “a” and “b”, respectively. Then, the two images are blended and stored in the variable “c” using the formula:

(10) c=(1−α)×a+α×b

**Figure 6.**
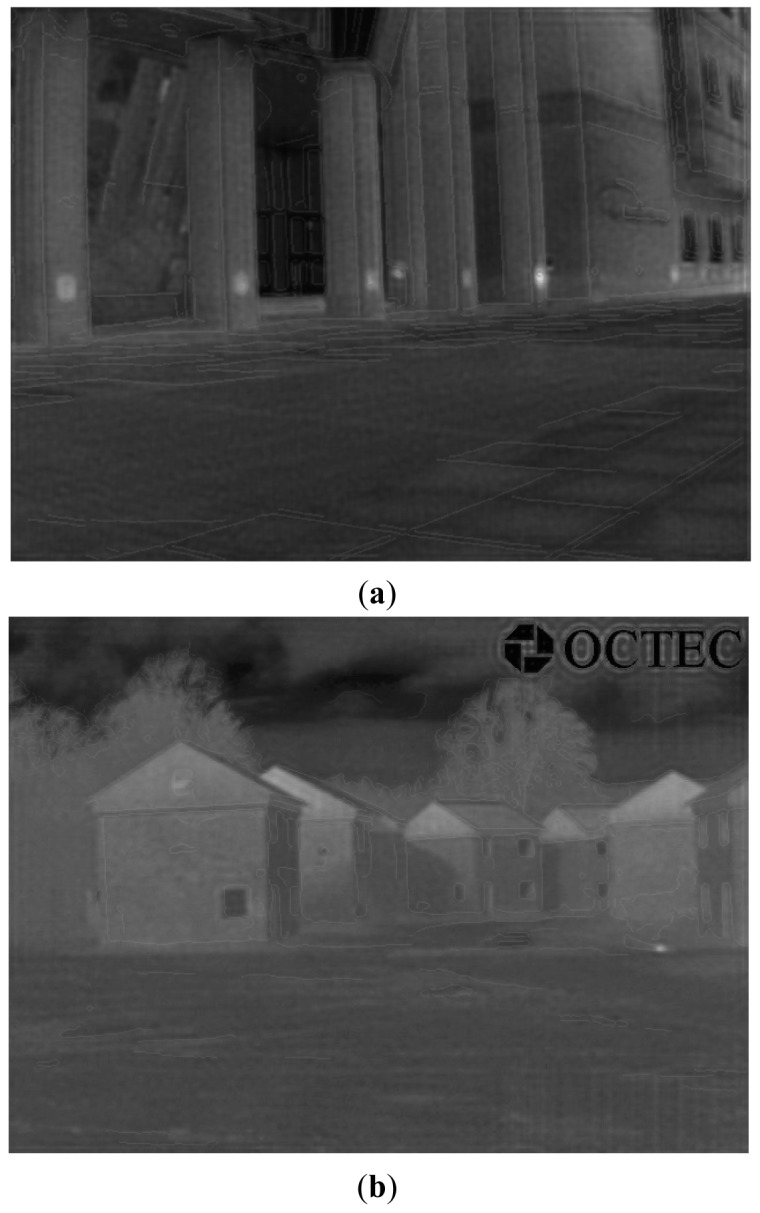
Blended images generated with both EO and IR images. (**a**) Blended result of IR Image 1; (**b**) Blended result of IR Image 2.

**Figure 7.**
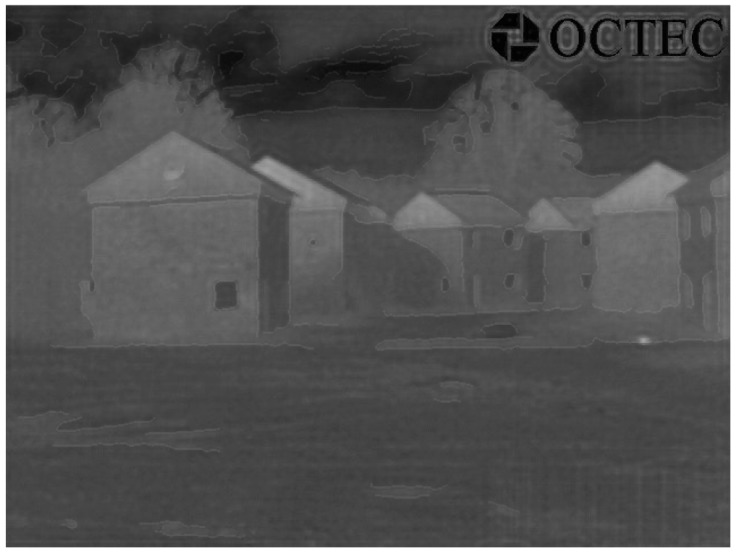
Blended result of IR Image 2 when only original IR image is available.

During this process, we first blend the transformed IR image with the original IR image, and then we blend the obtained result with the edge-detected image. Here, we fix the blending fraction's values to be 0.8 and 0.01, respectively. Figure 6 shows two blended images that are generated with both EO and IR images. Based on the same steps, Figure 7 shows a blended image that is generated when only original IR image is available, *i.e.*, with the edges extracted from the IR image. Seen from the obtained results, we can clearly see the seamless edge in the blended images and accurately distinguish objects at night via the superimposed edge, especially for objects with a similar temperature, and tiny parts of any object, which are difficult based on traditional object distinguishing approaches. However, in the intended applications, EO images and IR images are captured at different time, which may lead to problems in cases where the situation changes.

Now, we are able to answer the questions in Section 1 as follows:

1. We can clearly see the edge of each object, which will help distinguish objects at night. We also can see the edge of a tiny part of any object, which seems to be impossible based on previous methods. Moreover, we can see the edge of any color gas (e.g., smoke) or liquid and, in some particular cases, we can even see the edge of any colorless liquid or gas. Blending the transformed IR image (temperature information) with the original IR image makes the final IR image look “sharper” than that of the original IR image only.
2. Blended IR images have better quality over the superimposed images, because the blended images can make the edge seamlessly match with the original objects, and the reduced edge's value can ensure that the edge is not prominent in the blended image. In addition, in the superimposed result (Figure 5(b)), there are several strips at the bottom-right side, which is generated during the IR image transformation process. However, the linear blending process could overcome this problem and obtain a fused result mostly close to the original IR image, which is shown in Figure 6(b).
3. Generally speaking, if only IR images are available, we can use the same parameter settings to complete the proposed three-step framework (without registration) and obtain similar blended results as those of available EO images. However, the detected edge of an object, such as the edge of its shadow area rather than the real object, is not accurate, and it is impossible to detect the shape of smoke (gas) only based on the original IR image only (see results of “OCTEC” image).
4. Partial means objects we can only see in a kind of image sensors. For example, we could only see smoke in EO image sensors. Therefore, by adopting our proposed framework, there is nothing we can see partially if both EO and IR images are available. However, if only IR images are available, we may see something partially. For example, we can only see partial gas/smoke, because gas/smoke appears in the EO image (Figure 4(c)), whereas we can only see a fire point from blended “OCTEC” IR image (Figure 7) rather than generated gas/smoke. The purpose of adopting “gas/smoke” example is just for highlighting some properties of the proposed framework, it has no relation with intended applications.
5. If we have both EO and IR images, there is no “false alarm”. However, if only IR images are available, sometimes there are “false alarms”. For example, if we have both EO and IR images, we could blend the edge of the house (Figure 4(d)) rather than its shadow area onto the transformed IR images. Therefore, there is no false alarm. On the contrary, if only IR image is available, the blended edge is the shadow area of a house (Figure 4(e)) instead of the house itself. Obviously, this is a false alarm. The extraneous pattern means effects in fused images are not caused by objects appeared in these images. For example, the shadow area of the house is generated by sunlight (not appear in the image) rather than the house itself. Therefore, we regard the shadow area as a kind of extraneous pattern. By contrast, smoke in the EO Image 2 is generated by fire (light point) appeared in the EO image, thus smoke cannot be accounted as an extraneous pattern.
6. Comparing the results of both EO and IR images with that of only the IR image, our proposed framework can help accurately distinguish any object in the IR image, even for any tiny part of an object.

## 5. Conclusions

It is a challenge to accurately distinguish objects with IR images at night, especially objects with a similar temperature or tiny parts of any object. Therefore, in contrast to the traditional approaches used in image fusing, we proposed two novel approaches to achieve this purpose, which are superimposing the edge-detected EO image onto the transformed IR image, and blending the edge-detected EO image with the corresponding transformed IR image and the original IR image. On the one hand, simulation results showed that we could clearly distinguish objects, and even small parts of a single object, regardless of their temperature; on the other hand, simulation results showed that the blended approach has better performance than the superimposed approach Generally speaking, if only IR images are available, our framework could achieve similar performances to those of available EO images, except for some special objects or objects that have some extraneous patterns. On the contrary, we will consider how to utilize merits of IR images to improve EO images in the future, e.g., include objects' temperature information in EO images.
